# Leukocyte Trafficking and Hemostasis in the Mouse Fetus *in vivo*: A Practical Guide

**DOI:** 10.3389/fcell.2020.632297

**Published:** 2021-01-21

**Authors:** Andreas Margraf, Markus Sperandio

**Affiliations:** ^1^Institute of Cardiovascular Physiology and Pathophysiology, Walter Brendel Center of Experimental Medicine, Ludwig-Maximilians-University Munich, Munich, Germany; ^2^Department of Anesthesiology, Intensive Care Medicine and Pain Therapy, University Hospital Muenster, Muenster, Germany

**Keywords:** fetal development, intravital microscopy, leukocytes, neutrophils, platelets, neonatology, physiology

## Abstract

*In vivo* observations of blood cells and organ compartments within the fetal mammalian organism are difficult to obtain. This practical guide describes a mouse model for *in vivo* observation of the fetal yolk-sac and corporal microvasculature throughout murine gestation, including imaging of various organ compartments, microvascular injection procedures, different methods for staining of blood plasma, vessel wall and circulating cell subsets. Following anesthesia of pregnant mice, the maternal abdominal cavity is opened, the uterus horn exteriorized, and the fetus prepared for imaging while still connected to the placenta. Microinjection methods allow delivery of substances directly into the fetal circulation, while substances crossing the placenta can be easily administered via the maternal circulation. Small volume blood sample collection allows for further *in vitro* workup of obtained results. The model permits observation of leukocyte-endothelial interactions, hematopoietic niche localization, platelet function, endothelial permeability studies, and hemodynamic changes in the mouse fetus, using appropriate strains of fluorescent protein expressing reporter mice and various sophisticated intravital microscopy techniques. Our practical guide is of interest to basic physiologists, developmental biologists, cardiologists, and translational neonatologists and reaches out to scientists focusing on the origin and regulation of hematopoietic niches, thrombopoiesis and macrophage heterogeneity.

## Introduction

### Background

Developmental processes including formation and function of blood cells and organs in mammalian fetuses are still incompletely understood. Mortality in very low and ultra-low birth weight premature infants shows only modest improvements, remains very high (mortality in 23 week old premature infants 73 vs 67% 2009 vs. 2012, respectively), and is the leading cause of death for children under 5 years of age according to the World Health Organization (WHO), showcasing the lack of adequate research and translational endeavors (Callaghan et al., 2006; Stoll et al., 2015). Human preterm infants are at high risk for infections and bleeding complications. Coping with increasingly younger gestational ages at birth challenges the clinical field, demanding further experimental workup of developmental processes. Cord blood samples of premature infants or *in vitro/ex vivo* analyses of animal fetuses are the sources used so far to obtain information about developmental processes of the blood system and its consequences for bleeding, inflammation and development. So far, for organ development, radiologic imaging techniques, such as CT-scans, MRIs, or pathologists’ and anatomists’ workup of deceased fetuses are our most suitable source of information. In this practical overview, we describe an intravital microscopy (IVM) approach to observe the growing mouse fetus including yolk sac, focusing on the fetal vasculature and blood cells with a particular interest on leukocyte trafficking during inflammation and fetal platelet function.

### *In vivo* Imaging of Fetal Yolk-Sac Vessels and Organ Compartments

The fetal IVM model was developed to investigate functional maturation and development of blood cell populations and progenitors in the living mouse fetus and elucidate underlying mechanisms of the regulation of homing processes and cell-cell interactions in the fetus.

Due to lack of knowledge about *in vivo* fetal responses to inflammatory stimuli together with clinical findings regarding postnatal complications in preterm infants, we set out to study leukocyte recruitment and leukocyte-endothelial cell interactions in the developing mouse fetus demonstrating an ontogenetic regulation of fetal leukocyte function with diminished leukocyte recruitment in the yolk-sac and fetal skull during fetal development (Sperandio et al., 2013). Subsequently, we focused on platelet function and platelet-leukocyte interactions *in vivo.* We wanted to understand to what extent thrombus formation could occur in the fetal vasculature (Margraf et al., 2017). This is an important question, as premature infants exhibit a high incidence of severe bleeding complications. In addition, adverse outcomes of premature infants have been reported in those infants receiving transfusions of adult platelets (Margraf et al., 2019). Our results herein showed that young fetuses have difficulties to form thrombi, with platelet hyporeactivity due to diminished expression levels of integrin adaptor molecules and decreased platelet counts (Margraf et al., 2017). More recently, we could use our fetal model in additional applications related to developmental processes of different cell populations and organ compartments, including the development of monocytes/macrophages in the fetus (Stremmel et al., 2018a,b) and the impact of blood flow properties on the development of the fetal thymus (Moretti et al., 2018). Here, we provide a concise approach for the preparation and subsequent IVM observation of fetal blood vessels and microinjection into the fetal vasculature in the mouse, including techniques to image platelets and leukocytes as well as different organ compartments.

### Applications of the Fetal Intravital Microscopy Model

While examination of blood cell function is one of the major benefits of the fetal IVM model, it features a large variety of possible applications. These include vascular function and development, vessel distribution and reactivity, yolk-sac stability, as well as healing and regeneration processes. As IVM is well established in adult models of the mouse [e.g., cremaster muscle preparation (Sperandio et al., 2006), dorsal skinfold chamber (Lehr et al., 1993), vessel injury (Pircher et al., 2012)], experimental protocols, such as trauma-induced inflammation, endothelial damage and/or stimulation with pro-inflammatory agents (using LPS, fMLP, TNF-α etc.) can be transferred to the fetal *in vivo* model. Also, barrier-crossing of maternally administered substances can be traced, and intrauterine exposure to inflammatory stimuli can be mimicked.

### Comparison With Other Methods and Advantages of the System

In past decades, different approaches to examine the fetal blood system or organ compartments have been applied. Christiansen and Bacon used trans-illumination microscopy on completely exteriorized fetuses to analyze vessel patterns in the developing posterior limb of mouse fetuses (Christiansen and Bacon, 1961). Echtler et al. (2010) applied a model of prematurity, where fetuses were born through cesarean section, intubated and used for experiments. This model allowed simulation and studying of clinically relevant problems, such as ductus arteriosus closure, while the preterm infant was challenged through outside influences, representing a disease-state setting, yet not allowing analysis of a developmentally regular surrounding, such as the yolk-sac. Another approach used a partial incision of the uterus musculature and yolk sac in combination with a suture-glue-fixation to prevent fluctuation of amniotic fluid, while displaying the fetal cranium in a fixed position for further analysis of the developing brain (Ang et al., 2003).

Additional methods featured MRI-trans-sections, where any movement could cause artifacts and no detailed analysis of blood cell subpopulations can be acquired at this moment due to limitations in traceable probes as well as low sensitivity (Dhenain et al., 2001; Speier et al., 2008). Garcia et al. (2011) chose an *ex vivo* embryo culture method to gain insight into morphogenetic events in the developing fetus, while Laufer et al. (2012) used photoacustic imaging techniques for CD-1 mice to examine embryos *ex vivo* and *in vivo*. Boisset et al. (2011) developed an approach where *ex vivo* confocal image acquisition of the embryo aorta was performed in order to monitor hematopoietic and endothelial cells during development. Yanagida et al. (2012) used a model for the visualization of migrating cortical interneurons in which an exteriorized E16.5 fetus, attached to the umbilical vessels is positioned in agarose gel with or without gallamine triethiodide application and scalp removal. Other techniques equally rely on incision of the yolk-sac and placement of the fetus into a heating chamber for example filled with artificial cerebrospinal fluid (Yuryev et al., 2015). Another technique used a fully mobilized uterine horn in which the mesometrium was cut and ovarian vessels had been ligated. The preparation was then mechanically immobilized, fixed in low-melting agarose and the embryo accessed by pressing it against the uterine wall and imaging it through the wall using two photon microscopy (Hattori et al., 2020).

Experimental models for murine fetal *in vivo* imaging are surprisingly rare and existing models have limitations regarding optical resolution, imaging techniques, or surgical preparation procedures with unintentional harming of the fetus itself. Thus, a model to study physiologically relevant developmental aspects has been lacking so far. Our *in vivo* model allows rather long observation times and a less artificial surrounding for the fetus itself, which remains vital throughout the experiments. Our preparatory techniques also allow removal of minute amounts of blood for further analysis from fetuses as young as age E13.5 (out of 21 days of gestation), e.g., for FACS analysis.

## Materials

### Experimental Design and Level of Expertise Needed

#### Animals and Timed Matings

One major logistic effort lies within the requirement of pregnant animals. Thus, timed matings are needed to ensure adequate estimates of developmental age, which further needs to be specified through correlation of anatomical properties. In our setting, timed matings were conducted through two females and one male animal in the cage put together for one night. Depending on the specific need of pregnant mice we calculated with three to four cages per successful pregnancy. Influence of pheromones is minimized through spatial separation and hygiene precautions. For this purpose, female animals are placed in a separate room, while mating takes place in the room where the male mating animals are housed. After mating, animals are checked for plug-presentation, separated into plug positive and negative and placed back into the female room and a separate plug-positive room, respectively. Behavior, weight and change of abdominal configuration are checked daily. Prior to experiments weight, agility, and abdominal curvature are re-evaluated to prevent false positive pregnancies.

#### Choice of Anesthetics

For *in vivo* experiments involving muscular preparations and requiring stable images, a combination of ketamine and xylazine (both known to cross the placental barrier) is used, to reduce movement of the uterine musculature, while ensuring sufficient anesthesia, and analgesia for the animal.

#### Choice of Fluorophores and Antibodies

It is crucial for *in vivo* imaging to ensure sufficient image contrast, stability, and penetration depth. While the last two points are mainly influenced by preparational skills and technical setup, the image contrast relies on the appropriate choice of fluorophores and plasma markers. Equally, choice of antibodies is important when only a limited amount of colors can be imaged at once. Table 1 gives a list of fluorophores and antibodies we and others have used in fetal blood cell imaging.

**TABLE 1:** Antibodies, fluorophores, and markers used for *in vivo* imaging in the mouse fetus.

Fluorophore, antibody or genetic marker	Category	Used for	Advantage	Disadvantage	Expected consequences	Considerations	Citations
FITC (high MW)	Fluorophore	Thrombus induction; Plasma marker	Bright, good contrast	Photoreactivity limits application as regular plasma-marker	Phototoxicity leads to vessel-wall injury and thrombus formation	Phototoxic	Margraf et al., 2017
TRITC (high MW)	Fluorophore	Plasma marker	Almost no leakage		Labeling of plasma and passive visualization of fluorescence-negative blood cells		Honkura et al., 2018
Low MW dextrans (for example 70 kDa Texas-red dextran and 40 kDa FITC dextran)	Fluorophore	Endothelial permeability assessment	Leakage		Observation of plasma staining and time-dependent increase in perivascular fluorescence intensity		Lee et al., 2018
Acridine orange	Fluorophore	Leukocyte and vessel wall labeling	Crosses placental barrier	Vessel constriction, thrombus induction	Labeling of leukocytes and endothelial cells	Phototoxic	Sperandio et al., 2013
GpIbβ-X488 antibody	Antibody	Platelet labeling	Non-blocking	Expensive	Labeling of platelets and megakaryocytes		Margraf et al., 2017
PeCam (CD31)-antibody	Antibody	Endothelial cell labeling		Expensive	Labeling of platelets and endothelial cells		Cappenberg et al., 2019
Gr-1 antibody	Antibody	Neutrophil labeling		Expensive, not specific; depletion (?)	Labeling of neutrophils and other leukocyte populations	Transient expression in monocytes	Volmering et al., 2016
Ly6G antibody	Antibody	Neutrophil labeling	Specific	Expensive, depletion (?), blocking (?)	Labeling of neutrophils		Wang et al., 2012; Yipp and Kubes, 2013; Cunin et al., 2019
*Lyz2*-EGFP	Genetic	Neutrophil analysis	Good signal intensity	Unspecific	Observation of leukocytes		Sperandio et al., 2013
CX3CR1 GFP	Genetic	Monocyte/macrophage analysis		Unspecific	Labeling of multiple monocytic/macrophage populations and progenitors		Stremmel et al., 2018a,b
IVM Catchup	Genetic	Neutrophil analysis	Good signal	Specific for neutrophils	Labeling of neutrophils	Homozygous animals have been described as Ly6G-deficient	Hasenberg et al., 2015
*Cdh5*Cre	Genetic	Crossing with floxed stop reporter mouse (f.e. Rosa26-floxed stop fluorescent protein) line to create endothelial cell labeling within fetus and/or mother	Allows differentiating maternal and fetal vessels	Complex breeding schemes for fetal vs. maternal labeling; depending on mouse line unspecific HC-expression	Labeling of endothelial cells in either maternal vessels, fetal vessels or both, depending on breeding scheme.		Payne et al., 2018
Sca-1 GFP	Genetic	Hematopoietic stem and progenitor cell labeling	Good visualization of fetal circulation and organs	Unspecific	Labeling of progenitor cells and endothelial cells.		Ma et al., 2002a,b
CD41	Genetic or antibody	Platelet labeling		Unspecific	Labeling of platelets, megakaryocytes and various progenitor cells		Ferkowicz et al., 2003; Mikkola et al., 2003; Li et al., 2005; Zhang et al., 2007
Pf4tdRFP	Genetic	Platelet and megakaryocyte labeling	Allows visualization of platelets	Unspecific	Labeling of platelets, megakaryocytes and progenitor cells		Calaminus et al., 2012

#### Fetal Ages

The choice of different developmental stages for IVM analysis is important for the experiments and depends on goal, site of expected observational events, preparational skills, and experimental duration (also compare “Limitations”). The maturation state of the fetus can be assessed by classical anatomical features and size of the mouse fetus, as described by Kaufmann (2005).

#### *In vivo* Imaging and Duration of Experiment

**FIGURE 1:**
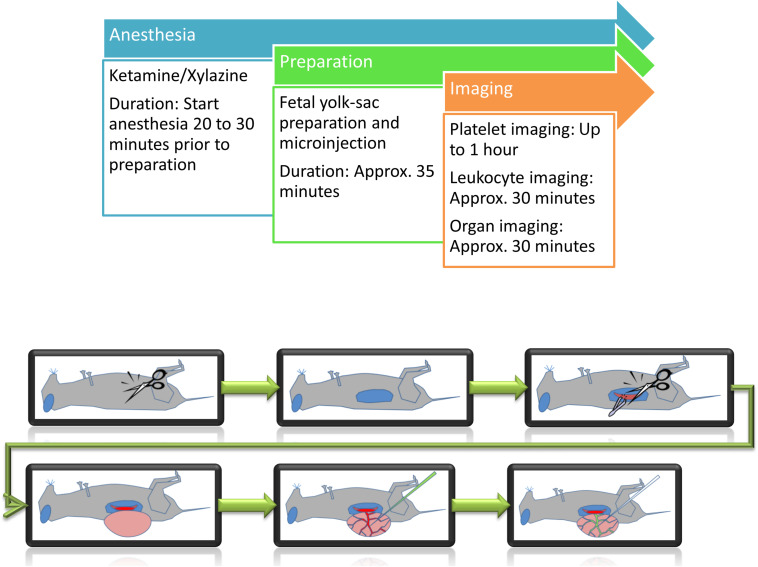
Schematic representation of preparation steps for fetal *in vivo* imaging. Following anesthesia, the yolk-sac is prepared for imaging and/or microinjection.

As any artificial manipulation can lead to serious consequences for the fetus, careful preparation and observation are necessary. Inflammatory stimulation or thrombus induction are harmful events occurring within the fetal vasculature, therefore limiting any subsequent experiments within the same fetus. Intravital imaging experiments were carried out for a maximum duration of 1 h per fetus (Figure 1).

#### Microinjection Volume Considerations

The developing murine fetus itself possesses only a small blood volume depending on the weight of the fetus (estimated 7–10% of the body weight). Thus, any injected substance will crucially influence circulatory mechanisms, cardiac output and vascular tone within the fetus (Russel et al., 1968). We observed that injection of volumes exceeding 5–10 μL strongly compromised the fetus and should therefore be avoided.

### Animals

#### Mice

Adult female (C57/Bl6; minimal age 12 weeks) and male mice are used for timed matings. Pregnant female mice are used for *in vivo* experiments. Through mating strategies and use of appropriate genetically modified reporter mice (Table 1), it is possible to generate different phenotypes in the fetus and mother. This might help to distinguish fetal from maternal structures (cells).

### Reagents

#### Anesthetic

Use a ketamine/xylazine mix (125 mg/kg bodyweight of ketamine; 12,5 mg/kg bodyweight of xylazine) in a volume of 0.1 ml per 8g bodyweight for anesthesia of the mother animal.

#### FITC-Dextran-Solution

Used to stain microvasculature and for phototoxicity-induced thrombus formation. Dissolve FITC-dextran in sterile injectable distilled water or sterile phosphate-buffered saline at a final concentration of 10%.

#### Acridine-Orange-Solution

Injectable *in vivo* dye capable of crossing the placental barrier. Dissolve at a concentration of 2 mg/ml in sterile phosphate-buffered saline. Prepare an injection volume of about max. 150 μl in a syringe for later administration (usually as needed, approx. 50 – 100 μl).

#### Microbeads

Used for *in vivo* blood flow velocity measurements. Ultrasonicate beads prior to usage. Dilute stock concentration of 1 × 10^10^ beads/ml per factor 10 to 100 according to wished study purpose. For injection into yolk sac vessels, dilute 1 μl of bead-solution in a total of 5 μl of sterile NaCl or PBS injection solution.

#### *In vivo* Superfusion Buffer

Classical superfusion solution for IVM experiments as reported earlier (Klitzman and Duling, 1979). Prepare solution I, containing 292.9 g of NaCl, 13.3 g of KCl, 11.2 g of CaCl2, and 7.7 g of MgCl2 in a total of 3.8 liters of deionized water. Prepare solution II, containing 57.5 g of NaHCO3 in 3.8 liters of deionized water. The *in vivo* superfusion buffer is then obtained by adding 200 ml of solution I into a two liter cylinder. Fill the cylinder then up to 1,800 ml with deionized water. Then add 200 ml of solution II to the cylinder. Mix the solution and add a gas-combination of 95% N2/5% CO2 using a foam-disperser. If needed, inflammatory stimuli (for example fMLP) can be added to the superfusion buffer.

### Equipment

#### Intravital Microscope

The intravital microscopic setup consists of an upright microscope, together with a motorized *xyz*-stage, which allows to save *xy*-positions and to move to a previously determined exact position again later throughout an experiment. Usage of inverted microscopes are not recommended as they can result in excessive pressure application onto the fetus and thus deterioration of blood flow in the field of observation. Equally, inverted microscopes do not allow for adequate superfusion of a fetus. For illumination different light sources (halogen lamp, Hg-lamp, stroboscopic flash lamp system, laser) are used together with a CCD-camera or photomultiplier tubes as appropriate for the type of microscopic technique (f.e., conventional fluorescence microscopy, multiphoton laser scanning microscopy, spinning disk microscopy and others, compare Table 3). The choice for usage of a specific microscope should depend on the research question in which either a fast image acquisition, long-term image stability or high resolution or tissue penetration are needed (see Table 3). To ensure optimal conditions for the animals during the IVM observation period, a heating pad and superfusion solution are used. The heating pad is placed below the mother animal to ensure adequate temperature of the maternal blood circulation which nurtures the placenta. Heating pad performance must be regularly controlled to guarantee appropriate environmental temperature. The superfusion solution is administered constantly with the use of a turning-pump/roller-pump. A polyethylene-tubing system is used to connect the superfusion system to the microscope objective. Temperature of the superfusion system must be adjusted to reach adequate temperature at the point of administration, thus measurements have to be performed directly at the objective. The temperature-controlled superfusion buffer in which the fetus is constantly immersed thus prevents cooling of the fetus itself. To guarantee a stable temperature of the superfusion buffer on the preparation, the superfusion buffer is continuously removed from the microscope stage through a small hole, connected to a vacuum pump reservoir (compare Supplementary Figure 1).

To keep the preparation in a fixed position, the mother animal is placed on a custom-made plexiglass animal stage, with the fetus positioned inside a petri dish (construction plan see Supplementary Figures 1–3), from which on one side, one part of the wall is removed to allow the fetus to lay inside the petri dish without the application of pressure or force onto it. The fetus is kept in place within the petri dish containing medical silicone-gel and the use of a custom made magnetic space-holder, which reduces the influence of the breathing movement of the mother-animal on the observation field. The space-holder contains a hole for microscopic access. A coverslip is placed on top of the fetus and used for observation. Care must be taken that the blood flow is not restricted by pressure application. Imaging techniques such as multiphoton-laser scanning-microscopy (MPLSM) are more sensitive to motion artifacts and thus require a higher degree of stabilization compared to conventional epifluorescence recordings. If the setup described herein is insufficient to achieve acceptable imaging conditions, users should consider the following options (also compare Tables 2, 3):

**TABLE 2:** Troubleshooting.

Step	Problem	Possible reason	Solution
Searching for the right microscopic image	The vessels are not satisfactory/too small/too big/none in the field of view.	Placement of animal, constricted blood circulation.	Place the mother animal on the abdomen and thus turn the whole preparation upside down, gaining access to other vessels within the yolk sac.
Microinjection	The vessel gets ruptured/it is difficult to get into the tissue.	Diameter of glass capillary is too big, not well grinded.	Try to choose a smaller diameter and make sure you check the grinded tip again before injection.
Microinjection	It is impossible to get the substance out of the glass capillary.	Diameter too small, blood clotting at the tip.	Grind the capillary to a larger diameter, try to apply pressure before the injection onto paper tissue to see if you can successfully inject, do not re-use used glass capillaries, as smallest amounts of blood can lead to closure of the tip.
Microinjection	I have a backflow into the glass capillary.	Pressure too low.	Adjust pressure to a higher level, making sure you keep a baseline level throughout puncturing and injection.
Microinjection	After injection and occlusion, tissue gets pulled out and the yolk-sac ruptures.	The cauterization device is sticking to the site of heat-occlusion.	Try to use only the tip of the cauterization device to occlude the vessel. Make sure you do it quick and precise. The bigger the area of heat-application, the more likely the yolk-sac will rupture.
Imaging	Using multiphoton microscopy, the resulting stacks are not showing the complete vessel in time lapse recordings.	The z-shift moves the vessel out of the focus. Shadow-effects by large amounts of erythrocytes contribute to poor penetration depth inside the vessel.	Increasing the z-stack size to levels sufficiently above and below the vessel of observation will prevent it from shifting out of the field of observation.
Thrombus induction	Mechanical vessel occlusion is not successful using local pressure application.	The stimulus is insufficient for studying thrombus formation.	Try to use a 8–0 suture in order to ligate the vessel locally. Keep the ligation for a longer time to increase flow restriction and vessel damage.
Imaging	Image quality is not satisfactory in the liver region.	Penetration depth is not sufficient.	Remove the surrounding tissue and liver capsule. Try to either ligate one of the ribs and pull it with a suture to the cranial direction, gaining access to the liver, or to carefully dissect part of the forming rib.

**TABLE 3:** Microscope applications.

Microscopic technique	Light source and detection method	Advantage	Disadvantage	Possible application	Fetal tissue
Conventional epifluorescence microscopy	Mercury bulb; digital camera.	Fast	Limited penetration depth, bleaching, and light scattering.	Assessment of leukocyte rolling, adhesion, and migration.	Yolk-sac, brain.
Spinning disc confocal microscopy	Laser; photomultiplier tubes and/or digital camera.	Fast with higher spatial resolution.	Does not reach penetration depth of MPSLM imaging.	Assessment of slow leukocyte rolling, adhesion, and migration.	Yolk-sac, brain, liver, and skin.
Conventional confocal microscopy	Laser; photomultiplier tubes.	High spatial resolution.	Slow	Analysis of expression patterns, assessment of stable structures or slow cell movement (migration).	Yolk-sac, brain, and liver.
Multi-Photon-Laser-Scanning-Microscopy (MPLSM)	Laser; photomultiplier tubes.	Label-free cell and tissue detection (second- and/or third-harmonic generation signals); high penetration depth; good spatial resolution.	Relatively slow, risk of laser damage depending on settings; motion sensitivity leads to artifacts and/or shifting of images.	Assessment of anatomical features; analysis of deeper organ compartments; migration; and laser-injury.	Yolk-sac, brain, liver, skin, bone marrow, and heart.

1. If insufficient perfusion of the yolk-sac is observed, try to reduce pressure exerted by the stabilization device. If necessary (especially in very old fetuses), try to only use a cover slip without fixation device and keep the cover slip in position by application of additional medical silicone-gel outside of the field of observation.
2. If the image is unstable, increase pressure while directly observing the microcirculation through the microscope, without affecting blood circulation within the yolk-sac or fetus. Generally, ensure the mother animal is not in contact with the cover slip or holding device otherwise breathing artifacts are transferred to the preparation. Also, it is helpful to model the silicone-gel against the fetus to ensure it remains within its position for the duration of the recording. Additional care must be taken to ensure adequate anesthesia, which might require re-injection of anesthetics depending on the duration of recording.

Recording of the *in vivo* observations is conducted via a digital recording system.

#### Generation of Micropipettes for Fetal Microinjection

For microinjection purposes, glass capillaries are being heat-pulled utilizing a vertical heat-puller. Employing a stereoscope, pulled glass capillaries (micropipettes) are placed in a grinding device and grinded to create a syringe-like tip (open tip diameter 1-2 μm), which allows easier penetration of blood vessels.

## Results/Procedures

A single experiment from the beginning of anesthesia until the end of image acquisition takes between one to one and a half hours for leukocyte imaging and two and a half hours for platelet function studies (Figure 1).

### Mouse Anesthesia and Surgical Procedure

Anesthetize the pregnant mother animal, using 100 μl narcotic cocktail per 8 g bodyweight. Administration of anesthesia should be i.m., rather than i.p., as effectiveness of i.p. injection might be influenced by the surgical procedure of the model, which requires opening of the abdominal cavity with potential leakage of the applied anesthetic drugs. In addition, i.p. injection might also harm the fetuses by misplaced injection. After administration wait for about 20 min, until the mouse is securely unconscious. Check anesthesia through a pain stimulus (e.g., compression of the foot-limb). Place the mother animal with its back on the heating pad. Disinfect the abdominal site of preparation with 70% ethanol. Shaving can be performed as needed. Make a lateral horizontal incision in the expected size of the fetus (approx. 1 cm) to open the abdominal cavity. Cauterize blood vessels from which bleeding might occur either before or during incision of the peritoneum.

### Preparation of Fetal Yolk-Sac Vessels

Localize the uterus horn and carefully grab it with blunt tweezers (Supplementary Movie 1). Try to hold on to muscle tissue of the uterine wall only, without grabbing the fetal body in order to prevent injury. This is most conveniently done in a region between two fetuses. Exteriorize the uterus. Make sure you prevent cooling and drying through administration of heated superfusion buffer (37°C) prior, during and after exteriorization. Incise the uterine musculature in a horizontal manner to reach to one vital fetus within its yolk sac. Place another incision in a vertical manner (90° to prior incision) to reduce pressure of the uterine musculature onto the placenta. Ensure to start the incision at the opposite site of the placenta, between two fetuses, where you can easily hold the uterus muscle tissue with blunt forceps, without harming the yolk sac. From there, extend the incision, using manually blunted microsurgery scissors. The uterus muscle fibers will start to contract and retract, giving access to the yolk-sac. It is very easy to puncture and/or rupture the yolk-sac while trying to cut through the uterine wall. Also, a high amount of pressure resulting from the contraction of uterine muscle fibers of the incised uterine horn, especially in older fetuses, can lead to the rupture of the yolk-sac and worsening perfusion. At this step, patience is necessary. Very often, the fetus within the yolk-sac finds its way out through the surgical opening of the placenta without external support.

**FIGURE 2:**
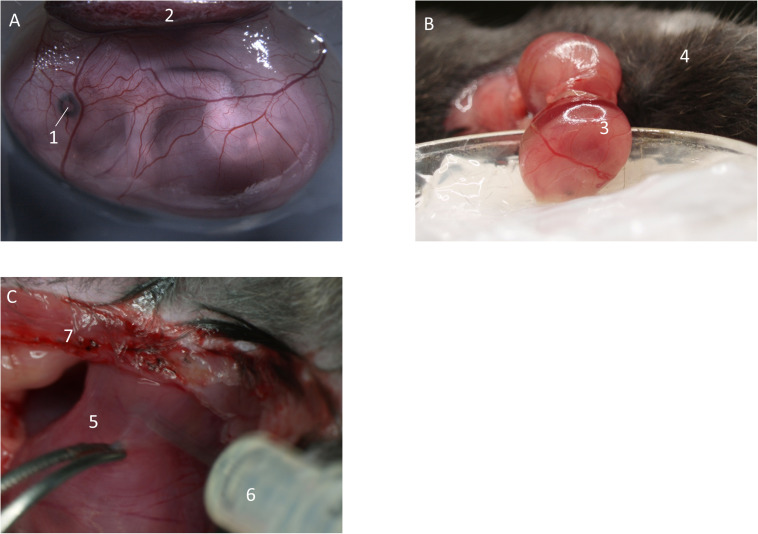
Preparation of fetal yolk-sac. **(A)** Preparation allows access to the yolk-sac microvasculature. Image from Margraf (2018). **(B)** Following incision of the uterus, the fetus inside the yolk-sac is carefully mobilized and placed in a modified petri dish filled with medical silicone gel and superfusion buffer. **(C)** If needed, inflammatory stimuli can be injected into the uterus by puncturing in-between two separate fetuses. (1) Eye of the fetus. (2) Placenta. (3) Fetus inside yolk-sac. (4) Mother animal. (5) Uterus horn in-between two fetuses. (6) Syringe. (7) Abdominal opening of the mother animal.

After the fetus is exteriorized (Figure 2A) and still inside the yolk-sac, the fetus is gently placed into a modified petri dish (5 ml) (Figure 2B), filled with silicone and warmed superfusion buffer solution. Lifting cannot be done by directly pulling on the yolk-sac, as it will easily rupture. Therefore, try to pull on surrounding uterine musculature, located next to the preparation site. It is also possible to load the fetus onto a pre-wetted cotton-stick and carefully mobilize it. It is important not to damage the placenta to decrease the risk of bleeding. After having secured the fetus within the yolk sac, the animal stage can be transferred to the IVM for imaging.

We have performed extensive imaging studies on yolk sac vessels (a) to elucidate the maturation of neutrophil recruitment during inflammation throughout mouse fetal development and (b) to investigate platelet function during fetal ontogeny. The following sections will describe how we approached these two processes by intravital imaging:

#### Studying *in vivo* Neutrophil Recruitment During Fetal Development

Depending on the purpose of the project, the preparation of the fetus/yolk-sac can be performed in unstimulated pregnant mice or pregnant mice in which the uterus has been pre-stimulated with proinflammatory agents (f.e., intrauterine LPS, fMLP, or TNF-α, Figure 2C) prior to imaging. If no proinflammatory stimulus is applied, the surgical procedure itself will cause a mild inflammatory response with some rolling and adherent leukocytes, which can be compared to trauma-induced injury as described in the mouse cremaster muscle (Sperandio et al., 2001).

Using reporter mice such as *Lyz2* EGFP mice (Faust et al., 2000) or Catchup IVM mice (Hasenberg et al., 2015), neutrophils can be visualized by their fluorescent signal. The yolk sac microcirculation does not need to be stained as the auto-fluorescence signal is bright enough for conventional fluorescence microscopy. Observation of rolling, adhesion and crawling of fluorescently labeled blood cells as neutrophils is then possible. In case no external stimuli are used, we see some rolling and adherent neutrophils, which increase in number with gestational age. For application of additional dyes or other agents into the maternal organism, a carotid artery catheter can be placed into the pregnant mouse before imaging. Injection of acridine-orange solution into the carotid artery will stain maternal and fetal leukocytes (placental passage!) and can be used as alternative approach in case reporter mice are not available.

Measuring of blood flow velocity:

Three different techniques can be applied to study blood flow characteristics during fetal ontogeny. The most precise and reproducible technique is the microbead based method. Overall, velocity is calculated as the displacement of a bead or cell from point a to point b during a pre-determined time interval, resulting in:

$$\text{Velocity}=\frac{\left(\mathrm{Distance}\,\mathrm{point}\,a\,\mathrm{to}\,\mathrm{point}\,b\right)\,\left[\mu \,m\right]}{\left(\text{Time}\right)\,\left[s\right]}.$$

1. Leukocyte based method: Using a flowing leukocyte in the center of the vessel, the movement of the cell is followed frame by frame. This gives you information on traveled distance over time.
2. Microbead based method: Microbeads are microinjected into the fetus and allowed to circulate prior to imaging. As described under (1) two consecutive frames can then be used and microbead displacement assessed over time.
3. Multiphoton-laser scanning microscopy-based line-shift determination of blood flow velocity can be performed as previously described (Dietzel et al., 2014).

### Studying Thrombus Formation and Platelet-Leukocyte Interaction in the Growing Mouse Fetus

Microinjection: Fill one grinded and prepared glass microcapillary with FITC-dextran, to a volume of approximately 5 μl (/beads/antibody-solution, respectively). Depending on the desired injection site, volume and opening diameter of the glass capillary, either manual pressure infusion, or a transjector-based approach can be chosen; equally, either manual puncturing or micromanipulator-based puncturing of the yolk-sac vessel can be performed.

Connect the microcapillary to the pressure-application tube. Hold the glass-capillary like a pencil, with the tip of your fingers, while using the other hand to stabilize. Make sure, to put the cauterization device close to the other hand (Supplementary Movie 1). Optional, you can hold it with your other hand (e.g., left, if right-handed), while puncturing the yolk-sac, to immediately occlude the injection-vessel in order to prevent bleeding out of the injected substance from the penetration point. Puncture a sidebranch vessel. Before doing so, observe blood flow characteristics, as it is crucial to choose a vessel which ensures distribution and flow into a bigger blood vessel. Rupturing and/or penetration of the yolk-sac is very easy during this step. Practice is necessary, while it can be helpful to know that due to the size of the glass capillary (depending on purpose of injection), tissue can be folded and pushed away during pressure application by the tip of the glass capillary (the bigger the tip diameter, the more difficult it will be to penetrate a vessel; yet the smaller the tip diameter, the more likely it will break or demolish the grinded tip). Once the applied puncturing-pressure is high enough, the tissue will allow access and the glass-capillary will slide into the blood vessel.

Applying constant injection pressure, administer the dye and/or antibody-solution into the vessel. Observe it through the stereo-microscope. Make sure to observe the level of the injection solution to prevent injection of air into the fetal vasculature. Thus, stop the injection right before air application and proceed to the next step, while maintaining a constant slightly positive pressure equivalent to the current intravascular blood pressure. It is easiest to manually adapt this pressure.

Remove the glass capillary from the vessel and immediately occlude the injection point with the electric-cauterization device. Be quick. If too slow, the injected solution will flow out of the vessel again. Additionally, it is important to minimize the applied heat/trauma using the cauterization device, to ensure sufficient blood flow in the surrounding vessels.

Leave the fetus for approximately 5–10 min in the dark room in warm superfusion buffer to guarantee circulation and thus distribution of the phototoxic dye and/or antibody-solution.

Imaging of the yolk-sac: Place the fetus onto the imaging-stage. Make sure not to rupture the connection between fetus and mother animal while positioning or moving the preparation. Transfer the preparation to the *in vivo* microscope setup. Choose appropriate light/filter/detector settings. For imaging choose a vessel away from the point of microinjection. Apply superfusion buffer throughout the experiment.

Thrombus induction: For thrombus induction, we recommend the phototoxic or laser-induced approach, yet depending on availability of techniques, also the mentioned other approaches can be used. Nonetheless, variation in results is greater in subsequently mentioned techniques.

#### Phototoxic Injury

For thrombus induction use phototoxic FITC-dextran as microinjection-solution. Perform imaging using a high intensity light source (e.g., mercury lamp). For FITC-Dextran, use a filter for excitation maximum: 490 nm, emission maximum: 520 nm. Observe one vessel (20–50 μm) for up to 1 h or until stop of flow occurs. Determine fluorescence intensity using histogram values. For our experiments we chose a camera exposure time of 10 ms. Of note, the field of view will be constantly illuminated by the light source throughout the experiment. Examine platelet adherence and vessel occlusion. Examine reflow-phenomena as an inverse correlate of thrombus stability for 10 min after complete occlusion of a vessel occurred. Once reflow appears, continue observation again for up to 1 h or until stop of flow occurs.

#### Laser-Induced Injury

For thrombus induction use 2-photon-imaging (Nishimura et al., 2006; Kamocka et al., 2010; Koike et al., 2011). Depending on laser-settings, use a small point laser scan in the level of the vessel wall. Create a vessel-wall injury using beam intensity slightly below apparent heat damage. Observe thrombus formation using time-laps stack acquisition. Movements in z-direction are difficult to outbalance. Thus, appropriate stack settings need to be chosen, allowing for a range of motion.

#### Chemical Injury

Prepare a 1 mm × 2 mm filter paper patch. Place the filter paper patch into FeCl3-Solution of desired concentration (e.g., 1%). Microinject GpIbβ-X488-antibody into the fetal vasculature and occlude the vessel (see above). A concentration of 0,1 μg/g body weight is recommended. Now apply the FeCl3-saturated paper patch onto the desired vessel under the stereomicroscope. Observe the surrounding area (borderline) of the patch-applied vessel for blood flow cessation. Remove the FeCl3-paper patch after a minimum time of 30 s and proceed quickly to the next step in order to observe the different steps of platelet-vessel-wall interaction. Perform imaging under the *in vivo* microscope using the appropriate filter sets for platelet observation. The forming clot can be noted as fluorescence enhancement at the site of adhering platelets. The antibody recommendation for imaging are to use FITC-fluorescence filter sets and exposure times between 200 and 400 ms depending on camera setup and excitation light source.

#### Platelet-Leukocyte Interactions

Utilizing antibody or genetic knock-in strategies to fluorescently label platelets and leukocytes (for example using a GFP-knock in for leukocytes and an Alexa649 antibody staining for platelets), platelet-leukocyte interaction can be quantified by counting double-positive (GFP+ and Alexa649+) cellular events and assessing rolling of leukocytes on adherent platelets. Both thrombotic (interaction of leukocytes with injury-related adherent platelets) and free circulating platelet-leukocyte aggregates can be enumerated. At early gestational ages no platelet-leukocyte aggregates can be observed as P-selectin and PSGL-1 expression levels are low. Transfusion of isolated, labeled adult platelets and/or leukocytes into older fetuses can help in dissecting cell- and maturation-specific phenotypes.

### Fetus Exteriorization and Organ Imaging

**FIGURE 3:**
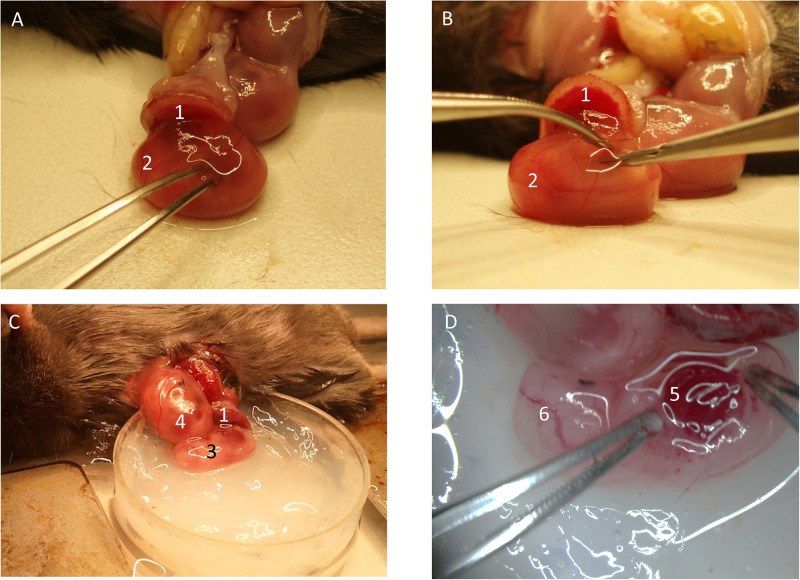
Preparation of fetal liver. **(A)** The yolk-sac is carefully opened. **(B)** The fetus is removed from the yolk-sac and **(C)** placed into the imaging-dish. **(D)** The liver region is prepared for imaging. (1) Placenta. (2) Fetus inside yolk-sac being mobilized. (3) Fetus without yolk-sac. (4) Fetus within yolk-sac. (5) Fetal liver. (6) Cranial region of fetus.

Carefully open the yolk-sac and exteriorize the fetus (Figure 3A). Ensure the umbilical vessels are still intact and not damaged. Remove disturbing tissue and liquid. Place the fetus inside a modified petri dish filled with superfusion buffer. Proceed with preparation and imaging as described below:

#### Skin

Place a cover slip onto the skin pattern you wish to image. Apply the fixation device. Perform *in vivo* imaging with constant superfusion. MPLSM can be used for deep tissue penetration.

#### Liver

Make a small incision within the posterio-lateral area of the fetus (Figures 3B,C), in an area where the liver is clearly visible through the thin skin (Figure 3D). From there, gently open the lateral side of the abdominal cavity of the fetus to display the fetal liver. If needed, carefully remove one of the forming ribs. It might be necessary to remove the liver capsule (Glisson’s capsule). The liver is a well perfused organ. Thus, preparation and manipulation within this area features high bleeding risk. Place a cover slip and the fixation device on top and transfer it to the imaging setup. MPLSM might hold best results due to its penetration depth.

#### Cranial Imaging

Carefully incise the skin in the head region (temporal region) and place a small silicon ring (approx. 0,5 to 1 cm diameter, depending on objective used for imaging) onto the top. Fill the ring with superfusion buffer for imaging of the cranial-window. Proceed to imaging.

### Blood Sampling for Flow Cytometry and Systemic Blood Cell Counts

Acquisition for systemic fetal blood cell counts requires exact volumes as the cell amount can be fairly low.

To collect fetal blood the following procedure can be applied: Completely exteriorize the fetus from the yolk-sac, dry the fetus and remove any amniotic fluid with soft cotton sticks, perform a lateral neck incision, and discard the first small droplet of blood. Then place a 5 μl collecting glass capillary onto the incision site, where the blood vessels are clearly visible. Observe blood collection through a stereomicroscope to ensure no surrounding tissue leakage is collected into the capillary. Transfer the collected sample into citrate solution (45 μl 0.11 M sodium citrate solution, pH 6.5). Prepare the sample by adding appropriate antibodies directed against the required cell subpopulation. Add microbeads of known quantity and volume for later volume determination. Transfer samples to the flow cytometer for analysis.

If functional assays with fetal blood cells are planned and higher blood volumes are necessary, the following procedure can be used: Remove the yolk-sac and completely exteriorize the fetus and wash fetus and placenta quickly once in PBS. Dry the fetus and place it into a large petri dish filled with modified Tyrodes-HEPES-heparin-buffer. Make sure to leave the placenta outside of the petri dish. Dissect the umbilical cord and cut the fetal head with sharp scissors. Leave fetuses to bleed for approx. 10 min. Remove the fetus from the petri dish and collect the suspended blood. Washing, adding of antibodies and flow cytometry analysis can then be performed.

## Discussion/Limitations

The described preparation and imaging techniques allow several different applications. Yolk-sac analysis and microinjection of fluorescent substances enable examination of vessel- and blood cell properties. 3D-image reconstruction will show fluorescently labeled vessels following microinjection of plasma markers in both the yolk-sac layer and the deeper layers of the fetal body. Thrombus induction with phototoxic dyes results in a negative contrast image, in which the forming thrombus can be noted as a reduction in fluorescence intensity compared to the plasma marker FITC dextran. A stable image with only slight movement at young gestational ages and more intense motion at older gestational stages, is expected. In the setting of thrombus formation, platelet adherence followed by complete vessel occlusion can be observed within 30 min to 1 h in only a reduced number of animals, depending on fluorescent dye concentration, excitation light source and gestational age of the fetus. Leukocyte observation will show slight fluorescence of the surrounding vessel wall with strong fluorescent leukocytes within the blood which show a reduced recruitment phenotype in young gestational ages. Additionally, multiphoton-microscopy can be used, to generate SHG and THG (second- and third-harmonic-generation)-signals. Most commonly, a simple 2-D fluorescence image acquisition can be used, to display blood cells circulating in the microvasculature of the fetal yolk-sac.

Our model holds great potential for *in vivo* imaging of developmentally regulated processes. Nonetheless, only a restricted range of developmental stages can be selected for yolk-sac experiments, as insufficient size and the onset of circulation at young stages and yolk-sac involution processes at older stages of fetal development limit the practicability of such preparations. In accordance with this, our model is most suitable for *in vivo* experiments between developmental stages E13 and E18. Yet, with further manual skills and practice, also younger animals can be used (E9.5 onward). For older fetuses (E18 and E19), opening of the yolk-sac in the required region will facilitate vessel imaging also at this stage of development. In particular, a small incision in the cranial temporal region will allow access to the fetus for cerebral imaging purposes. Fetuses below the age of E13 are difficult to prepare due to the watery structure and lacking stability of the organism. Therefore, exteriorization of the fetus at this stage is accompanied by high fetal mortality.

**FIGURE 4:**
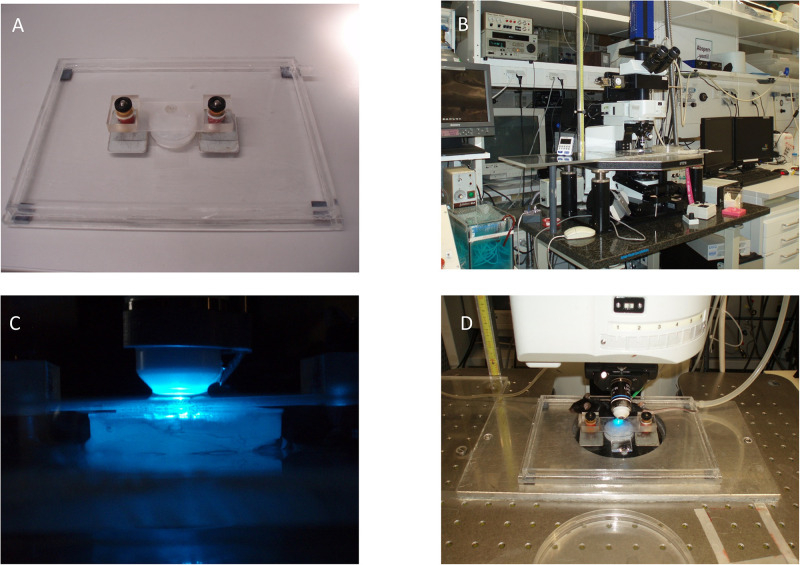
Exemplary fixation and microscope setup. **(A)** Microscope stage with magnetic spring-counter-weighed holding device. **(B)** *In vivo* microscopy setup including water bath and roller pump (lower left of the image). **(C)** Epifluorescence setup with superfusion-buffer needle attached to the objective. **(D)** Placement of the fetus and mother animal for imaging.

As the yolk-sac is exteriorized and a counter-weighed fixation-device is used to stabilize the imaging setup, the experimenter must be aware of the possible influence of these manipulations on basic physiologic processes (Figure 4). Additional pitfalls include usage of large beads at high concentrations to study blood flow velocity. This could result in blockage of circulatory routes. Modulations in the cardiovascular and hemodynamic parameters of the mother animal will impact placenta perfusion and thus affect the fetus. Consequently, strict control of vital parameters and physiologic conditions not only for the fetus itself but also of the mother animal is needed throughout the experiment.

Using antibodies, labeling substances or fluorescent protein-expressing genetically modified animals (compare Table 1), this model has a wide range of applications. In this context, one must be aware of the difficulties of genetically engineered reporter mice as well as antibody labeling, as classical cell markers might be developmentally regulated and only appear late during fetal life or are temporally expressed in other cell types during fetal ontogeny. Examples include CD41 for platelets (Mikkola et al., 2003).

Ongoing research focusses on developmental aspects of leukocytes, platelets, megakaryocytes, macrophages, endothelial, and progenitor cells. Whereas general developmental aspects (platelet-hyporeactivity and leukocyte hyporeactivity) are of great interest for neonatologists and physiologists, more specific research questions will have to address the precise underlying mechanisms for each uncovered phenotype. Not only therapeutic options in the neonatal period depend on this, but knowledge with regard to aging and maturation could potentially be obtained and translated to the understanding of adult malignancies or modification of therapies. Even though this model holds great advancements for the scientific community, the performing researcher must be aware of inherent ethical conflicts. Ethical considerations with regard to the mentioned techniques must include not only general animal welfare regulations but should place special focus on the protection of unborn life, hindering unnecessary application of such invasive methodology without justifiable scientific and translatable purpose.

Summarizing, this model offers a powerful technique to study *in vivo* processes in the developing mouse fetus and advance our understanding of basic physiologic and disease-related processes during ontogeny.

## Data Availability Statement

The original contributions presented in the study are included in the article/Supplementary Material, further inquiries can be directed to the corresponding author/s.

## Ethics Statement

The animal study was reviewed and approved by the responsible authorities at the Regierung von Oberbayern.

## Author Contributions

AM and MS developed the protocol, composed the manuscript, and performed experiments including image acquisition. Both authors contributed to the article and approved the submitted version.

## Conflict of Interest

The authors declare that the research was conducted in the absence of any commercial or financial relationships that could be construed as a potential conflict of interest.

## Supplementary Material

Supplementary Figure 1 Measurements of imaging stage.

Supplementary Figure 2 Measurements of holding device.

Supplementary Figure 3 Measurements of incised petri dish.

Supplementary Figure 4 Exemplary imaging setup.

Supplementary Movie 1 Preparation and microinjection of fetal yolk-sac vessels.

Supplementary Movie 2 Conventional epifluorescence recording of leukocyte rolling (green) and recruitment in the yolk-sac of a *Lyz2*-GFP positive fetus. Autofluorescence of tissue allows for identification of blood vessel lumen.

Supplementary Movie 3 MPLSM recording of a *Lyz2*-GFP positive fetus (green) injected with TRITC-dextrane (red-gray) and an Alexa649-coupled non-blocking GpIb antibody (blue).
