# Supplementary material for: Leukocyte Trafficking and Hemostasis in the Mouse Fetus in vivo: A Practical Guide
Source: Front Cell Dev Biol. 2021 Jan 21;8:632297. doi: 10.3389/fcell.2020.632297 (PMC7858264; doi:10.3389/fcell.2020.632297)
Supplement: Supplementary Figure 1 — Measurements of imaging stage. [file Data_Sheet_1.PDF]

Supplemental Figure 1: Measurements of imaging stage.

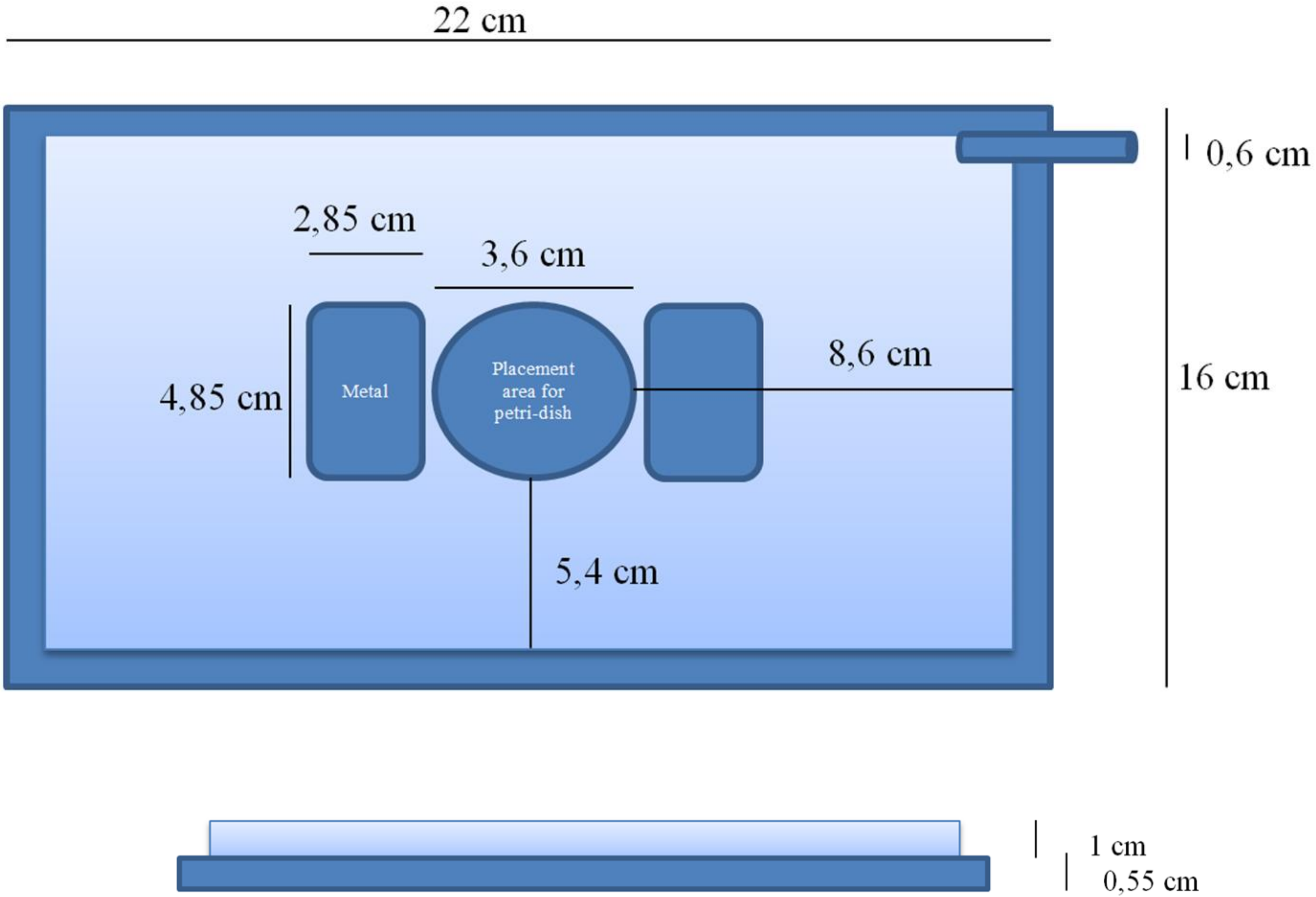

Supplemental Figure 2: Measurements of holding device.

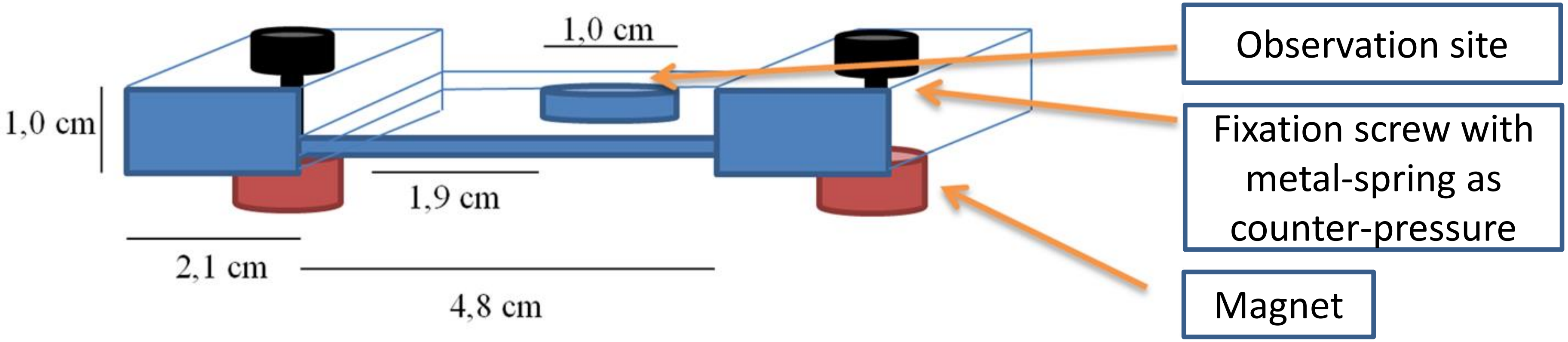

Supplemental Figure 3: Measurements of modified petri dish.

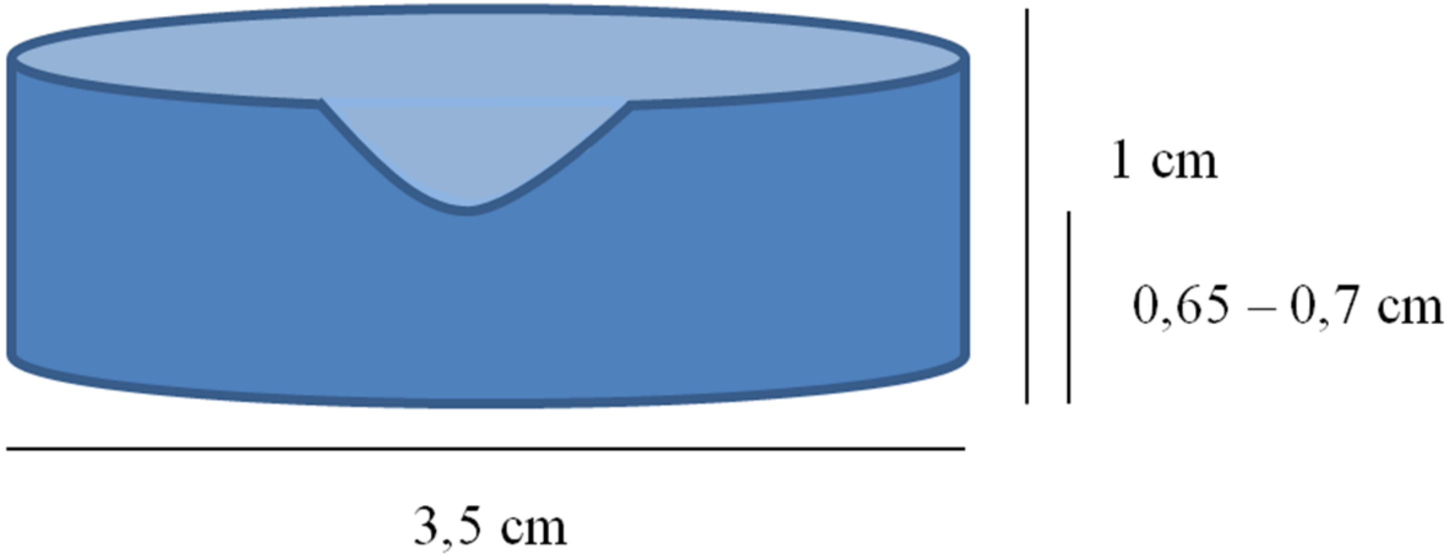

Supplemental Figure 4: Exemplary imaging setup.

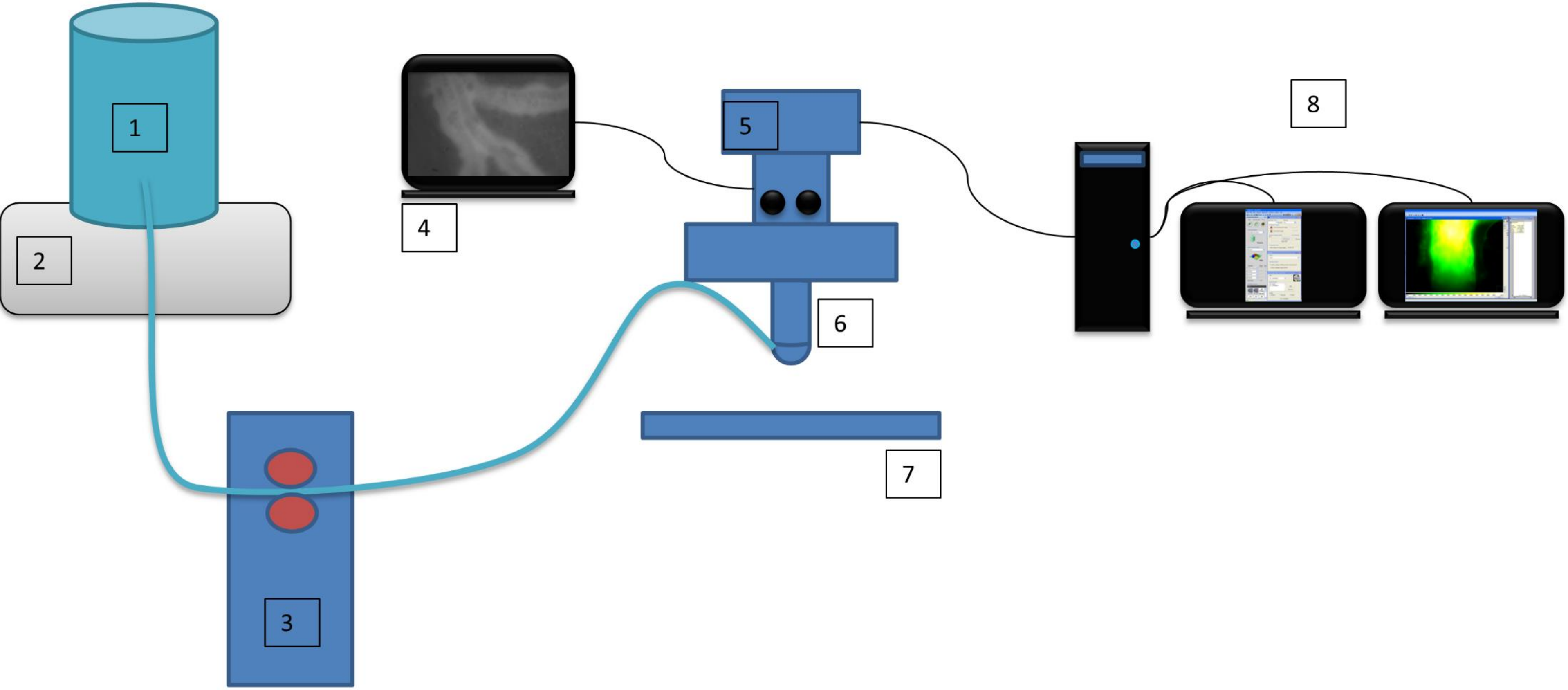

Figure legend:

1: Superfusion buffer reservoir; 2: Heater; 3: Roller-pump and waterbath; 4: TV-Screen; 5: CCD-Camera; 6: Microscopy setup with superfusion-tube connected to the objective; 7: Microscope stage; 8: Computer workstation
